# Intelligent analysis and measurement of semicircular canal spatial attitude

**DOI:** 10.3389/fneur.2024.1396513

**Published:** 2024-09-16

**Authors:** Mi Zhou, Jiesheng Mao, Xiaoqing Li, Yanjun Li, Xiaokai Yang

**Affiliations:** Third Affiliated Hospital of Shanghai University (Wenzhou People’s Hospital), School of Medicine, Shanghai University, Wenzhou, China

**Keywords:** MRI, deep learning, semicircular canal, orientation, medical image segmentation

## Abstract

**Objective:**

The primary aim of this investigation was to devise an intelligent approach for interpreting and measuring the spatial orientation of semicircular canals based on cranial MRI. The ultimate objective is to employ this intelligent method to construct a precise mathematical model that accurately represents the spatial orientation of the semicircular canals.

**Methods:**

Using a dataset of 115 cranial MRI scans, this study employed the nnDetection deep learning algorithm to perform automated segmentation of the semicircular canals and the eyeballs (left and right). The center points of each semicircular canal were organized into an ordered structure using point characteristic analysis. Subsequently, a point-by-point plane fit was performed along these centerlines, and the normal vector of the semicircular canals was computed using the singular value decomposition method and calibrated to a standard spatial coordinate system whose transverse planes were the top of the common crus and the bottom of the eyeballs.

**Results:**

The nnDetection target recognition segmentation algorithm achieved Dice values of 0.9585 and 0.9663. The direction angles of the unit normal vectors for the left anterior, lateral, and posterior semicircular canal planes were [80.19°, 124.32°, 36.08°], [169.88°, 100.04°, 91.32°], and [79.33°, 130.63°, 137.4°], respectively. For the right side, the angles were [79.03°, 125.41°, 142.42°], [171.45°, 98.53°, 89.43°], and [80.12°, 132.42°, 44.11°], respectively.

**Conclusion:**

This study successfully achieved real-time automated understanding and measurement of the spatial orientation of semicircular canals, providing a solid foundation for personalized diagnosis and treatment optimization of vestibular diseases. It also establishes essential tools and a theoretical basis for future research into vestibular function and related diseases.

## 1 Introduction

**Figure 1:**
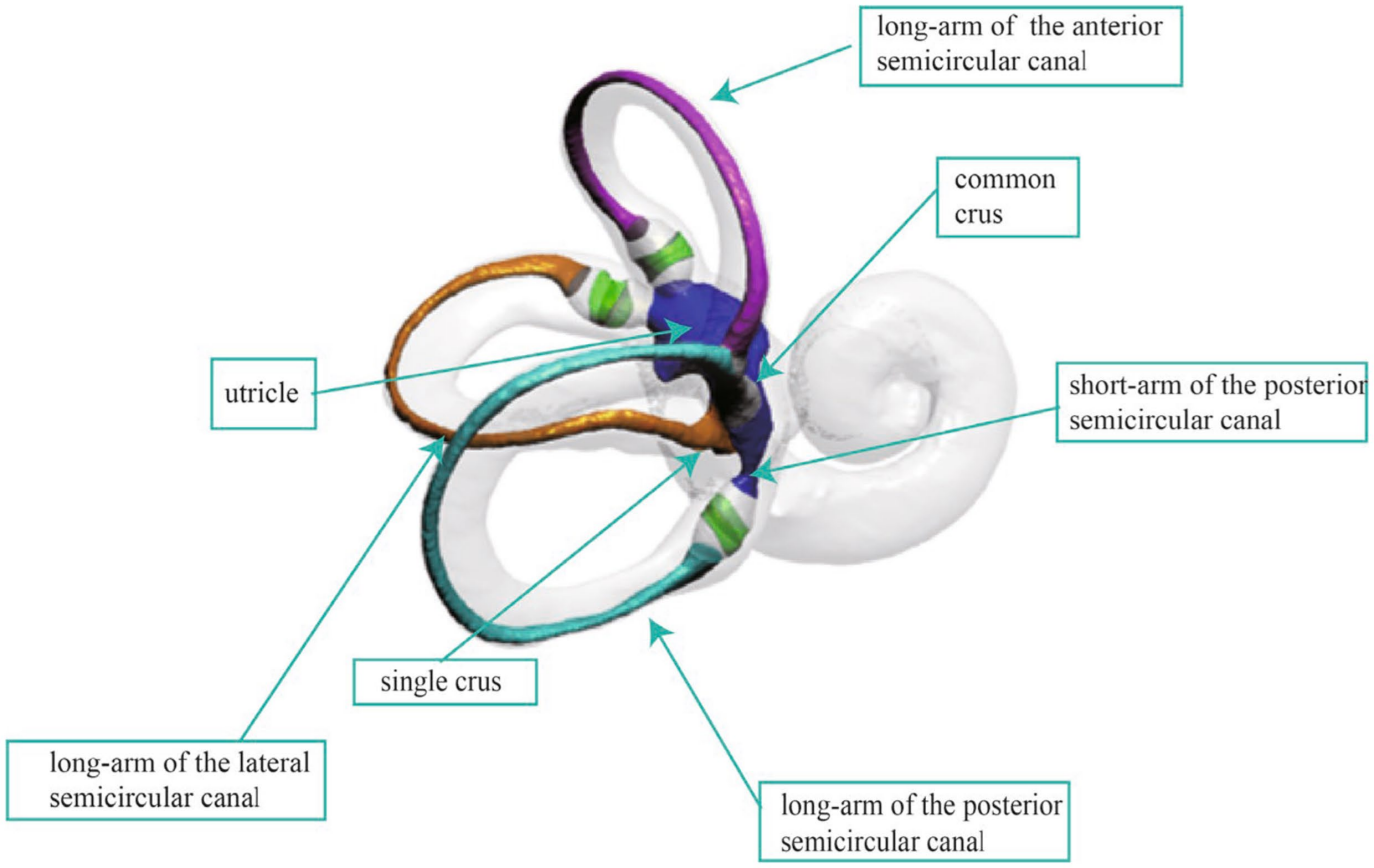
The anatomical structure of the inner ear.

The human semicircular canal system, an integral part of the vestibular organ, is essential for maintaining balance and spatial orientation. As shown in Figure 1, the semicircular canal system consists of three canals, each oriented nearly perpendicular to the others, enabling the encoding of angular acceleration within the plane of each respective canal (1). The inner ear is densely encased and shielded by the temporal bone, making it challenging to obtain the complete spatial morphology of the three semicircular canals through anatomical means. However, the spatial morphology of the semicircular canals directly influences the flow of endolymph and the biomechanical response of the hair cells in the crista ampullar. Notably, the diagnosis of Benign Paroxysmal Positional Vertigo (BPPV) and the positional changes required for otoconia repositioning heavily rely on the spatial orientation of the semicircular canal system (2–4). Consequently, accurately acquiring the spatial orientation information of the semicircular canals is fundamental to understanding and researching vestibular function and disorders.

The development of medical imaging techniques, such as CT and MRI, has opened up new possibilities for studying the spatial orientation of the semicircular canals in humans. Unlike the limitations of tissue sectioning, micro-CT, or magnetic resonance microscopy (MRM) examinations that only capture immediately adjacent structural landmarks, clinical CT or MRI examinations are able to establish a spatial coordinate system from cranial bony landmarks. Additionally, this method is non-invasive to the human body. Acquiring the spatial morphology of the semicircular canals through imaging techniques has become a research focus. This process can be broadly divided into two parts: segmenting the semicircular canals and measuring their orientation.

Manual segmentation remains the most prevalent method for segmenting semicircular canals. However, owing to the intricate and complex structure of the semicircular canals, operators require a comprehensive understanding of the anatomical structure of these canals. Manual segmentation is a laborious and time-consuming process, and is not a feasible technique to measure the spatial attitude of the semicircular canals within the clinical environment. Therefore, automated segmentation approaches are necessary. This involves the utilization of Convolutional Neural Networks (CNNs) for segmenting the semicircular canals. Increasingly, researchers both domestically and internationally are employing deep learning algorithms for segmenting the inner ear structures, with many scholars dedicating efforts to this area of study (5). Jeevakala S and colleagues proposed a U-Net-driven MaskR-CNN method to detect and segment the inner ear, thus generating accurate bounding boxes for the inner ear (6). Heutink F et al. conducted automatic cochlear segmentation on 123 CT image sets, achieving a Dice coefficient of approximately 0.90 (7). Hussain and team utilized an automatic context-based cascaded U-Net model to segment the labyrinth on temporal bone CTs, enabling automatic inner ear segmentation within seconds (8). Vaidyanathan and colleagues trained a 3D U-Net model for automatic inner ear segmentation on 944 MRI scans, which performed well on a clinically validated dataset with a dice similarity coefficient (DSC) of 0.8768 (9). Wu et al. utilized an improved 3D U-Net for the automatic segmentation of semicircular canals, incorporating spatial and channel attention mechanisms in their study, achieving a Dice coefficient of 92.5% on the test dataset (10). Li Zhenhua and team, based on the anatomical characteristics of the temporal bone, achieved automatic cochlear segmentation using the UNETR model, obtaining accuracy close to manual segmentation (11). The development of medical image processing technology has witnessed a shift from traditional methods to machine learning and deep learning algorithms. However, challenges such as insufficient segmentation accuracy and poor noise resistance in small targets persist. More importantly, the above research does not achieve recognition of the spatial attitude of the semicircular canals.

Recognizing the spatial attitude of semicircular canals involves acquiring the semicircular canal planes and establishing a three-dimensional coordinate system. Due to a certain degree of torsion exhibited by the semicircular canal planes, with the anterior canal displaying the most pronounced torsion, employing different methods to define the canal planes may lead to varying measurement results. The literature reports diverse methods for measuring semicircular canal angles, with variations in the location and number of measurement points. These methods primarily include multi-point plane equation fitting (12, 13), rotational coordinate plane method (14, 15), centerline plane equation fitting and Fourier function fitting (16), and three-point plane equation method (17, 18), each with its distinct advantages and limitations. The centerline fitting method for semicircular canals is the most accurate, but it is also the most complicated technique. Calculating the plane equation and the plane inclusion angle by taking three-point coordinates within the semicircular canal is straightforward; however, it is susceptible to errors when selecting the point locations. Furthermore, the spatial coordinate systems employed for measuring semicircular canal attitudes, as reported in the literature, also vary. The Frankfort and Reid coordinate systems are the most used plane systems for measuring canal attitudes (19, 20). The Reid plane is defined by the auriculares point, which is the center of the external acoustic meatus orifice, and the orbital point, located at the inferior margin of the orbit. Similarly, the Frankfort plane is defined by the bilateral porion (the midpoint on the upper margin of the external auditory canal), which are the anterior margin of the external auditory meatus, and the left orbital point. In the anatomical position, both the Reid and Frankfort planes are parallel to the lateral plane. Wu et al. (21) found that the Yang’s plane constructed by the top of the common crus and the lowest point of the eyeball was parallel to the Frankfort plane, contrary to Aoki et al.’s assertion that the plane constructed by the top of the common crus and the midpoint connecting the centers of both eyeballs was parallel to the Frankfort plane. The study of semicircular canal spatial attitudes based on MRI data cannot establish the Reid and Frankfort planes due to the limited scanning range and the difficulty in determining bony landmarks. As a result, these studies are typically limited to morphological measurements of the angles between the semicircular canals. Yang’s plane discovery provides an available reference coordinate system for studying the spatial orientation of semicircular canals based on MRI data.

In this study, we proposed a novel method that integrates deep learning with a 3D skeletonization algorithm to extract the centerlines of the semicircular canals from MRI images. Furthermore, we conducted a comprehensive analysis of each point on the centerlines using a custom-designed semicircular canal-eyeball spatial coordinate system. Our automatic segmentation model, built upon the nnDetection framework, enables simultaneous segmentation of the semicircular canals and eyeballs, laying a solid foundation for subsequent analysis (22). The method employs a 3D skeletonization algorithm to precisely extract the centerlines of the semicircular canals, which is crucial for determining their spatial orientation. Moreover, this study introduces a pioneering three-dimensional coordinate system that utilizes a plane formed by the tops of both sides of the common crus and the bottom of the eyeballs as the lateral plane. This innovative approach effectively addresses the challenge of establishing a reliable spatial coordinate system in MRI data, which often lacks clear bony landmarks (23).

To ensure robustness against noise and outliers, computational efficiency, and clear geometric interpretation, the singular value decomposition (SVD) method was applied to fit planes to the centerlines (24). The method leverages the properties of eigenvectors to achieve optimal plane fitting. Lastly, curve equations are employed to accurately model the three-dimensional spatial curves of the semicircular canals’ centerlines. A thorough analysis of key features, such as the normal vector of the semicircular canal plane, curvature, and torsion, is performed to enable intelligent assessment of the spatial orientation of the semicircular canals.

The goal is to create a precise mathematical model of the spatial orientation of the posterior, anterior, and lateral semicircular canals by averaging the unit normal vectors of each semicircular canal plane.

## 2 Materials and methods

### 2.1 Patients and data acquisition

This study included 115 patients with normal inner ear structures confirmed by MRI from January 2014 to December 2023, comprising 48 males and 67 females, with an average age of 50 years, ranging from 15 to 74 years old. Specific inclusion and exclusion criteria were used in this study. Inclusion criteria included clear display of semicircular canals and eyeball bottoms without artifacts, and successful segmentation of the complete semicircular canal in subsequent steps. Exclusion criteria included the presence of local lesions that may affect the anatomical structure of the semicircular canals, and any abnormal head structures.

A 1.5T superconducting MRI system from Siemens, a standard head coil, and the 3D-CISS sequence were used to examine the inner ear. The scanning parameters were TR: 6.0 ms, TE: 20.7 ms, FOV: 135 × 180, matrix: 256 × 192, thickness: 0.7 mm. Image processing and modeling of the original image data were exported from PACS and saved in DICOM format. The images were loaded into 3D Slicer version 5.3.0, and the 3D-CISS sequence was automatically exported and saved in the nii.gz format. The image spacing was 0.3515625 × 0.3515625 × 0.699983 mm.

### 2.2 The segmentation of semicircular canals and eyeballs

#### 2.2.1 Data set preprocessing

We employed 3D Slicer software to annotate the inner ear images. The annotation process was conducted using the built-in annotation boxes provided by the tool, enabling precise labeling of the inner ear structures. Upon completion of the annotation, the software automatically saved and generated files in the nii.gz format, ensuring data integrity and compatibility. Furthermore, we applied an identical processing approach to the eyeballs, separating them from the surrounding tissue to facilitate accurate segmentation. To optimize the accuracy of the segmentation labels, we employed a technique that involves generating a surface model using the marching cubes algorithm. This process includes label augmentation and voxel mining. The resulting surface model is then reimported as refined labels. This approach enhances the precision of the segmentation results by improving the quality of the input labels.

#### 2.2.2 Model architecture and model training

nnDetection is a self-configuring framework for 3D (volumetric) medical object detection that can be applied to new datasets without manual intervention. It systematizes and automates the configuration process for medical object detection tasks. The method adapts itself automatically to arbitrary medical detection problems while achieving results on par with or superior to the state-of-the-art. For implementation details and usage instructions, the nnDetection project code is available on GitHub.^1^

**Figure 2:**
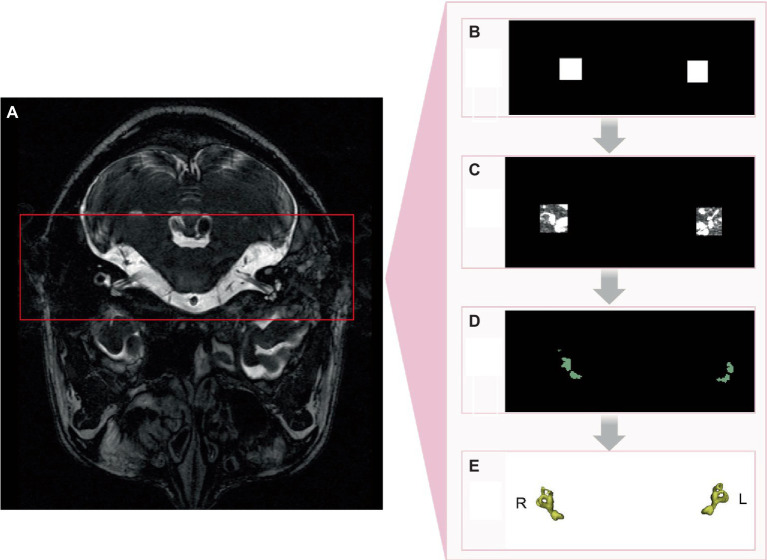
nnDetection implementation process. **(A)** Original image. **(B)** Identification of region of interest. **(C)** Semicircular canals within region of interest. **(D)** 2D segmentation of semicircular canals. **(E)** 3D segmentation of semicircular canals.

By utilizing the nnDetection framework for segmentation and target identification, a classical network model was primarily selected to mitigate the gradient vanishing issue caused by the accumulation of network depth, avoid noise interference from unrelated areas, and thus more effectively improve the segmentation accuracy. The configuration process for nnDetection involved extracting data fingerprints (representing the input data) and employing heuristic rules to determine rule-based parameters. Subsequently, full-resolution and low-resolution models were trained using five-fold cross-validation, where the data were divided into five subsets, and the model was trained and evaluated on each subset. After the training phase, the empirical parameters were determined by summarizing the predictions of the five models obtained through cross-validation, and the final predictions were generated (Figure 2).

#### 2.2.3 Segmentation evaluation

To assess the accuracy of the deep learning-based segmentation, we compared the model’s output (predicted segmentation masks) against the manually annotated ground truth masks (reference segmentation masks) using the Dice similarity coefficient (DSC). The DSC is a widely used metric for evaluating the spatial overlap between two segmentations, ranging from 0 (no overlap) to 1 (perfect overlap).

For each MRI scan in the test set (a subset of the data not used for training or validation), we computed the DSC between the predicted and reference segmentation masks for the semicircular canals and eyeballs separately. The DSC was calculated as follows:


$$DSC=\frac{2\left|X\cap Y\right|}{\left|X\right|+\left|Y\right|}$$

where X represents the set of voxels labeled as the structure of interest (e.g., semicircular canals) in the predicted segmentation mask, Y represents the set of voxels labeled as the same structure in the reference segmentation mask, and |X ∩ Y | denotes the number of voxels common to both sets. |X| and |Y| represent the total number of voxels in each set.

The DSC was computed for each structure in each test scan. The mean and standard deviation of the DSC across all test scans were then calculated to provide an overall assessment of the segmentation accuracy. By comparing the predicted segmentation masks against the manually annotated reference masks using the DSC, we quantitatively evaluated the performance of the deep learning-based segmentation approach in delineating the semicircular canals and eyeballs.

### 2.3 Analysis of the spatial attitude of the semicircular canal

#### 2.3.1 Obtaining the centerline of each semicircular canal

**Figure 3:**
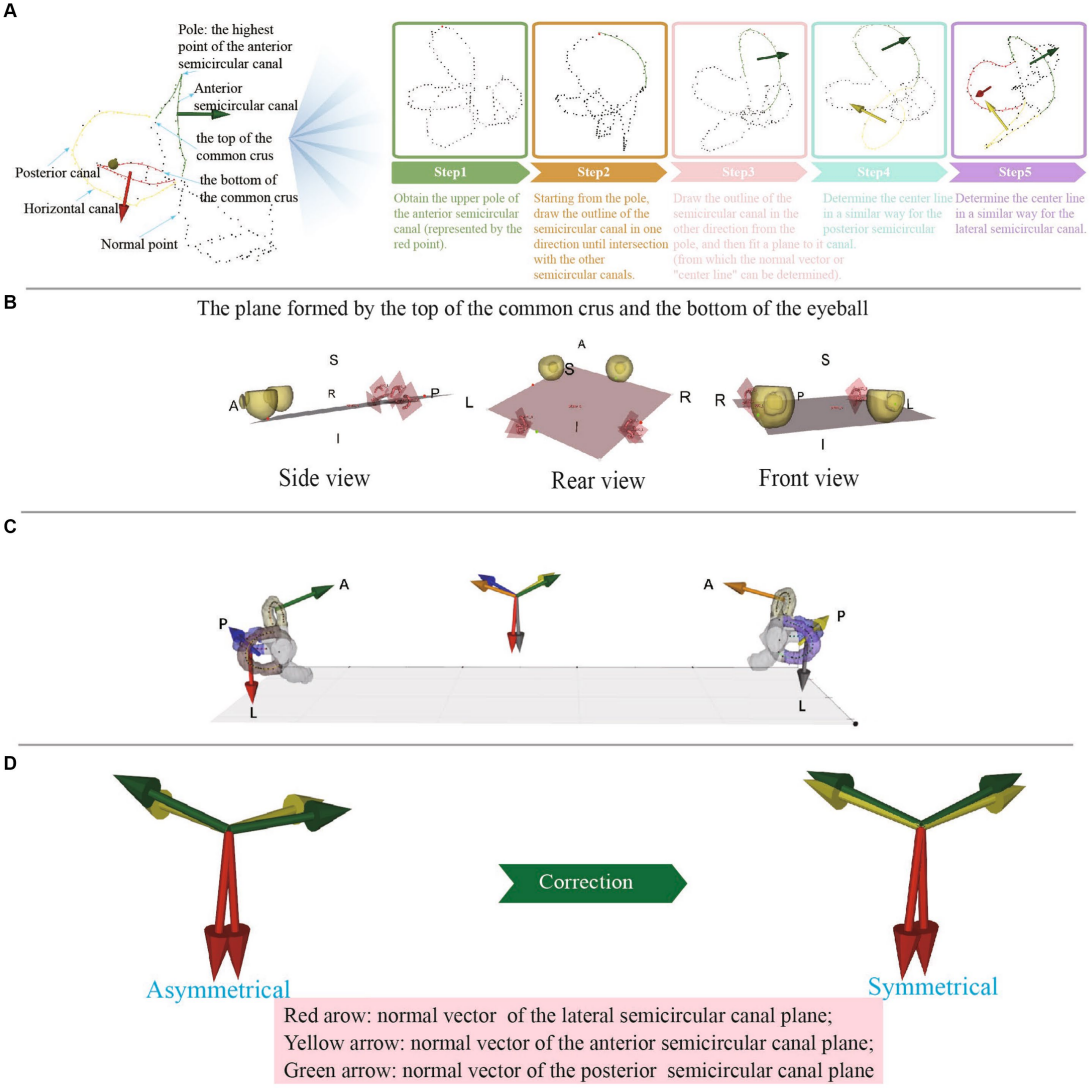
**(A)** The process of obtaining the center line of each semicircular canal. **(B)** The plane formed by the top of the common crus and the bottom of the eyeball. **(C)** The normal vectors of different semicircular planes are represented by arrows of different colors and are synchronized at the center of the semicircular planes and at the centers of mass, which allows visual observation of the semicircular plane normal vectors. **(D)** Anomalous data can be detected by observing whether the normal vectors of the left and right semicircular plane are symmetrical.

There were five main steps to obtain the centerlines of the semicircular canals: (1) A three-dimensional skeletonization algorithm read the binary image of the semicircular canals to extract their centerlines; (2) Given the left–right symmetry of the semicircular canals, the centerlines were divided into left and right semicircular canals based on the center of mass; (3) Utilizing the characteristics that the posterior semicircular canal is located most posterior, the anterior semicircular canal is located at the top, and the lateral semicircular canal is located most lateral, the centerline poles of the corresponding semicircular canals were obtained first, serving as key points for identifying each semicircular canal; (4) A point-to-point distance matrix was established and, according to the distance relationships, crossing points, ordinary points, and discrete points could be identified. A crossing point was defined as having more than two adjacent points in the near field; a discrete point was defined as having no adjacent points in the near domain; ordinary points were two adjacent points in the near field. The nearest neighbor threshold was set to 2 based on the actual situation; and (5) Starting from the poles of each semicircular canal centerline, adjacent points in both directions were connected until a crossing point was encountered, constructing an ordered circuit of the semicircular canal centerlines. The two ends of this circuit were the crossing points, and they were allowed separation into the lateral semicircular canal, posterior semicircular canal, and anterior semicircular canal centerlines. The identification of the lateral, posterior, and anterior semicircular canals was considered robust due to the reliable nature of the semicircular canal centerline poles (Figure 3A).

#### 2.3.2 Defining the standard spatial coordinate system

To measure the spatial attitude of a semicircular canal, we needed establish a three-dimensional spatial coordinate system. We used the following two steps:

1 Constructing the lateral plane based on anatomical landmarks in the image coordinate system

The MRI image data were represented in a three-dimensional array, with the width, length, and height corresponding to the X, Y, and Z axes, respectively. The *X*-axis ran from left to right, the *Y*-axis from posterior to anterior, and the *Z*-axis from inferior to superior.

First, we established the initial fundus plane using the lowest point of the eyeballs (the point with the lowest *Z*-value) and the tops of the two common crus (Figure 3B). Then, the plane was finely adjusted: (1) The distance between each point on the eyeball and the plane was calculated using the mathematical formula of the plane and the coordinate values of each point. (2) The point with the greatest distance was then chosen as the lowest point of the eyeball in the standard coordinate system. (3) The lowest point of the eyeballs (one point) and the tops of the two common crus (two points) were connected to form a lateral plane.

2 Determining the coordinate axes

To establish the spatial coordinate system, we determined the coordinate axes. With the head adjusted to a forward-facing position, the semicircular canal-eyeball plane served as the lateral plane, with its normal vector being the *X*-axis, whose positive direction pointed upward. The line connecting the tops of the left and right common crus was designated as the *Z*-axis, which was the normal vector of the sagittal plane, with its positive direction pointing rightward. The normal vector of the coronal plane was assigned as the *Y*-axis, which was the cross product of the *Z* and *X*-axes, with its positive direction pointing from posterior to anterior (Figure 3C).

#### 2.3.3 Semicircular canal attitude measurement

Firstly, the normal vector of the semicircular canal plane needed to be converted from the original coordinate system to the standard space coordinate system established according to the semicircular canal eyeball. It could be obtained by calculating the angle between the normal vector of the semicircular canal plane and the axis of the standard space coordinate system.

Secondly, the average value of the normal vector of the semicircular canal plane in the standard space coordinate system was calculated by summing the vectors and calculating the standard vectors.

Thirdly, the angle between each normal vector and the average normal vector was calculated to evaluate the deviation range of the normal vector. At the same time, to observe whether there were errors in the normal vector of the semicircular canal plane, each normal vector of the semicircular canal plane was represented by arrows with lines, and it could be intuitively judged whether the left and right were symmetric and whether there was deviation.

The semicircular canal and eyeball were displayed synchronously, the semicircular canal center line was marked with different color, and the semicircular canal center line was fitted with a spline line, which could be used to intuitively judge whether there were errors in the binary label (Figure 3D). The semicircular canals demonstrated left–right symmetry. By observing the symmetry of the semicircular canal plane normal vectors on the left and right sides, the accuracy of the measurement could be determined.

##### 2.3.3.1 Semicircular canal plane point selection

We employed B-spline curves to fit the centerline of the semicircular canal, as they provide a flexible and accurate way to represent complex curve shapes. The B-spline curve equation was defined as follows: Given n + 1 control points P0, P1, P2, …, Pn, which are used to define the direction and boundary range of the spline curve, the definition of a k-order B-spline curve with n + 1 control points is:


$$p\left(u\right)=\overset{n}{\underset{i=0}{\sum }}P_{i}B_{i,k}\left(u\right)$$

where $B_{i,k}\left(u\right)$ is the i-th k-order B-spline basis function corresponding to the control points, k ≥ 1; u is the independent variable.

In Python, the ‘splprep’ and ‘splev’ functions from the scipy.interpolate library provide a B-spline parametric representation of the curve and its derivatives. The smoothing factor, denoted as s, plays a crucial role in determining the curve’s behavior. A larger value of s results in a smoother curve, while a smaller value of s causes the curve to more closely approximate the given data points. By default, s is set to 0, which means the curve will pass exactly through all the data points. However, if s is assigned a positive value, the splprep function will attempt to smooth the data points, allowing the curve to deviate slightly from the exact points while maintaining a certain degree of smoothness. This flexibility enables the curve to balance between closely following the data and being smoother.

Curvature characterizes the degree of bending of the curve in the osculating plane including how much a curve deviates from being a straight line, while torsion describes the degree of twisting of the curve away from the osculating plane. The curvature and torsion of a three-dimensional curve could be calculated using its parametric representation.

The parametric equation of a three-dimensional curve was:


$$r\left(t\right)=\left(x\left(t\right),y\left(t\right),z\left(t\right)\right)$$

where t is the parameter. The following formulas were used to calculate the curvature κ and torsion τ:

Curvature κ:


$$\kappa \left(t\right)=\frac{∥r^{\prime }\left(t\right)\times r^{\prime \prime }\left(t\right)∥}{∥r^{\prime }\left(t\right){∥}^{3}}$$

Torsion τ:


$$\tau \left(t\right)=\frac{\left(r^{\prime }\left(t\right)\times r^{\prime \prime }\left(t\right)\right)\cdot r^{\prime \prime \prime }\left(t\right)}{{\left||r^{\prime }\left(t\right)\times r^{\prime \prime }\left(t\right)|\right|}^{2}}$$

where r’(t) was the first-order derivative of the position vector with respect to the parameter t, r”(t) was the second-order derivative, and so on.

##### 2.3.3.2 Semicircular canal plane point selection

Although the centerlines of each semicircular canal had been obtained, fitting the entire length of the semicircular canal centerline to the semicircular canal plane was not appropriate. The points of the semicircular canal centerline near the ampulla would deviate significantly from the plane, and these points needed to be removed when fitting the plane. The manual selection of points for fitting planes to the semicircular canal centerline is an extremely tedious and time-consuming task. To address this issue, we developed a fully automated process that relied on the analysis of torsion and curvature along the semicircular canal centerline. By using B-spline curve fitting to fit the semicircular canal centerline and calculating the curvature and torsion of each point, an understanding of the spatial attitude of each point on the semicircular canal centerline could be obtained. Our approach began by standardizing the torsion and curvature data, which was followed by the application of the Z-Score method for outlier detection, using the criterion of 2 standard deviations (2 SD) for defining outliers. Finally, the positions of the identified anomalies were returned. This step enabled us to accurately pinpoint the points with abnormal torsion and curvature at both ends of the centerline, allowing for their removal and the extraction of the final semicircular canal centerline. By applying this automatic procedure, we aimed to obtain accurate and reliable centerline representations of the semicircular canals, which served as the basis for subsequent analysis and measurement of their spatial orientation and morphology.

##### 2.3.3.3 Semicircular canal plane fitting

This study utilizes the SVD method to fit the semicircular canal plane. The SVD method has strong robustness to data noise and outliers when fitting planes in three-dimensional space, meaning that even if there was a certain amount of noise or outliers in the data, the SVD method could still obtain a relatively stable fitted plane (Figure 3B).

The specific steps were as follows:

1. Represent the point set on the semicircular canal centerline as matrix A, with each column representing the coordinates of a point.
2. Calculate the centroid of the point set, i.e., the mean of all point coordinates, for data centering, making the mean zero.
3. Center the point set to obtain matrix B. Subtract the centroid coordinates from each column of matrix A, so that the point set is centered at the origin.
4. Perform SVD decomposition on matrix B to obtain three matrices U, S, and V. Matrix V is called the right singular matrix and is composed of the eigenvectors of the sample. The row vectors represent the principal directions of the data, and the last row corresponds to the smallest singular value, representing the direction of the smallest variance in the data, i.e., the normal vector of the fitted plane.
5. Take the last row of matrix V as the normal vector of the fitted plane.

##### 2.3.3.4 Semicircular canal spatial attitude analysis

For the posterior, anterior, and lateral semicircular canal, respectively, the unit normal vectors were summed for all the same semicircular canal planes and the unit normal vector was obtained as the average normal vector of the corresponding semicircular canal from each subject.

For instance, if semicircular canal S has n local planes with corresponding unit normal vectors V_1_, V_2_, …, Vn, then the average normal vector of S was given by:


$${\vec{V}}_{avg}=\frac{\vec{v_{1}}+\vec{v_{2}}+\ldots +\vec{v_{n}}}{\left|\left|\vec{v_{1}}+\vec{v_{2}}+\ldots +\vec{v_{n}}\right|\right|}$$

where ||·|| represents the norm of a vector.

This normalization method ensured that ${\vec{V}}_{avg}$ remained a unit vector and integrated directional information from local planes across the semicircular canal. The resulting average normal vector represented the overall spatial orientation of the canal. The same technique for calculating semicircular canal attitude measurement was used as described above.

It is important to note that the normal vectors of the left and right semicircular canal planes were not perfectly symmetrical. However, if a visually apparent discrepancy existed, it may have been caused by various factors, including inaccurate segmentation, imprecise centerline extraction, or inappropriate point selection range. In such cases, manual correction was necessary to address the specific cause of the discrepancy (Figure 3D).

## 3 Results

### 3.1 Training results and segmentation results

**Figure 4:**
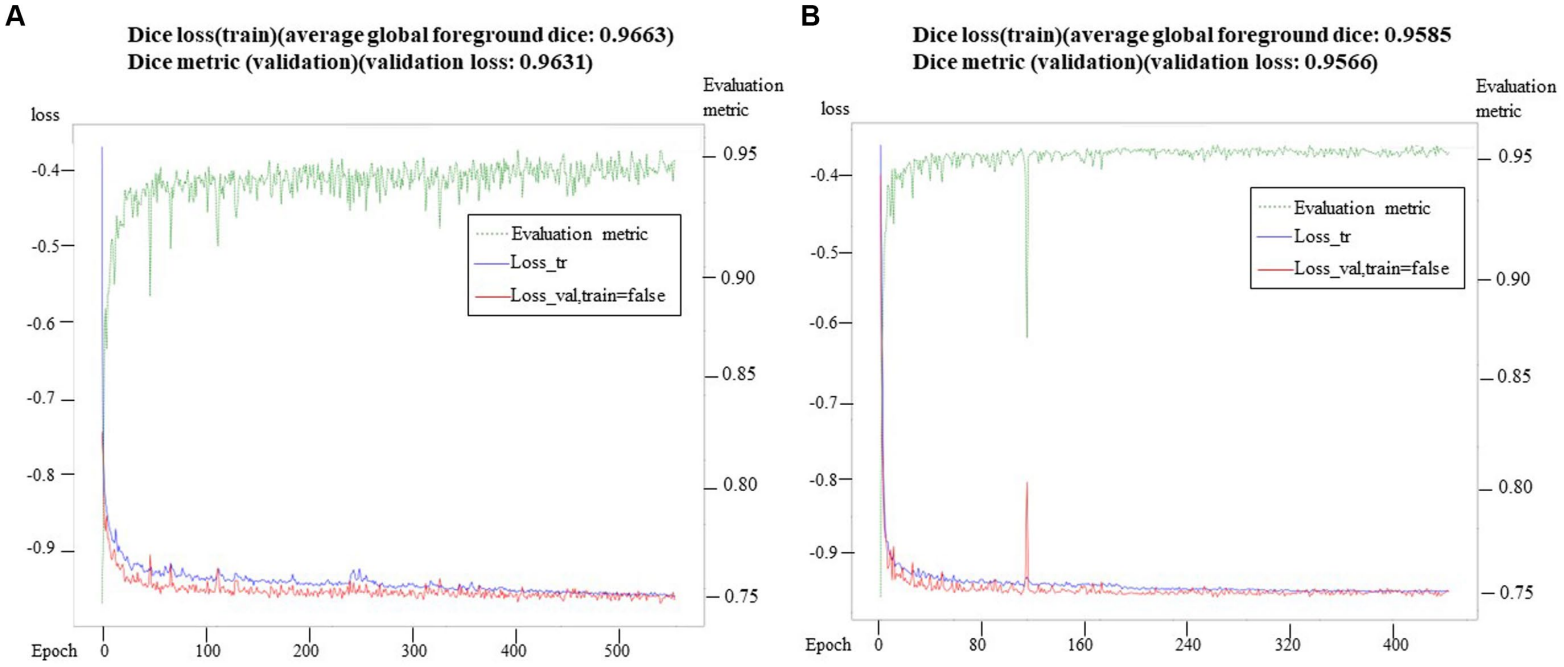
Training loss and verification metric. In **(A)**, the training loss and verification index were observed in 500 epochs, while in **(B)**, the training loss and verification index were observed in 388 epochs. At the same time, the verification index showed stable convergence in the whole optimization process, and there was no sign of over-fitting.

To evaluate the segmentation performance, we utilized the average Dice coefficient (DC), which provides a quantitative measure. The maximum validation values of the average Dice coefficient were recorded after 500 epochs and 388 epochs, yielding values of 0.9585 and 0.9663, respectively. These results indicated the effectiveness of the segmentation model. We preserved the best-performing model for further validation and testing on independent datasets. It is worth noting that the loss curve displayed stable convergence, reaching its minimum value in the final iteration. Additionally, the validation index consistently showed convergence throughout the optimization process, without any signs of overfitting. The total training time for the model was approximately 18.5 h. In addition to segmenting the inner ear, we also successfully segmented the eyeballs. Eyeball segmentation proved to be a relatively simpler task compared to inner ear segmentation. The same model that performed well in inner ear segmentation was employed for eyeball segmentation, yielding satisfactory results. The Dice loss of the model training and the change of the corresponding Dice index on the retention verification set are shown in (Figure 4). Abscissa indicated the number of training rounds, and ordinate indicated the size of the loss value. The change in the loss curve reflected the stability of the model. It could be seen that with the increase of epochs, the model tended to be stable when 60 epochs were trained, and the training loss was close to the verification loss, indicating that the network achieves the expected training effect.

### 3.2 Measurement of the spatial attitude of the semicircular canals

In this study, the X, Y, and Z coordinate axes represented the normal vectors of the lateral, coronal, and sagittal planes, respectively. As a result, we could deduce the angles between the semicircular plane and the lateral, coronal, and sagittal planes by examining the cosine of the direction of the unit normal vector of the semicircular plane. In other words, the direction angle of the unit normal vector of the semicircular plane provided information about its angle with the lateral, coronal, and sagittal planes (Tables 1, 2). The direction angles of the unit normal vectors for the left anterior, lateral, and posterior semicircular canal planes were [80.19°, 124.32°, 36.08°], [169.88°, 100.04°, 91.32°], and [79.33°, 130.63°, 137.4°], respectively. For the right side, the angles were [79.03°, 125.41°, 142.42°], [171.45°, 98.53°, 89.43°], and [80.12°, 132.42°, 44.11°], respectively.

**Table 1:** The mean normal vectors for the left semicircular canals.

Left	*X* axis	*Y* axis	*Z* axis
Anterior	0.1704	−0.5637	0.8082
Lateral	−0.9844	−0.1743	−0.0230
Posterior	0.1852	−0.6511	−0.7360

**Table 2:** The mean normal vectors for the right semicircular canals.

Right	*X* axis	*Y* axis	*Z* axis
Anterior	0.1903	−0.5795	−0.7925
Lateral	−0.9889	−0.1484	0.0099
Posterior	0.1716	−0.6746	0.7180

Based on the normal vectors of the semicircular canal planes, the angles between the semicircular canal planes could also be calculated (Table 3). The three ipsilateral semicircular canals were not completely perpendicular to each other, especially since the ipsilateral posterior and anterior semicircular canals made an angle of nearly 100 degrees. The posterior semicircular canal on one side and the anterior semicircular canal on the opposite side lay in the same plane at an angle of 7 degrees. Similarly, the right and left lateral semicircular canals lay in the same plane at an angle of 2.4 degrees. In particular, the angle between the posterior semicircular canal and the sagittal plane was 44 degrees. The angle between the lateral semicircular canal plane and the lateral plane was 10 degrees.

**Table 3:** The angles between semicircular canal planes.

Semicircular canal plane	Semicircular canal plane	Dihedral angle (degrees)
Left_A	Left_P	102.28
Left_A	Left_L	95.52
Left_A	Right_A	108.72
Left_A	Right_P	7.08
Left_A	Right_L	93.31
Left_P	Left_L	92.93
Left_P	Right_A	6.44
Left_P	Right_P	95.4
Left_P	Right_L	96.73
Left_L	Right_A	92.12
Left_L	Right_P	94.26
Left_L	Right_L	4.05
Right_A	Right_P	101.84
Right_A	Right_L	96.05
Right_P	Right_L	92.47

P, posterior semicircular canal; A, anterior semicircular canal; L, lateral semicircular canal.

The angle between the normal vector of each semicircular canal plane and the mean normal vector of the semicircular canal planes could reflect the range of variation in the normal vectors of the semicircular canal planes (Table 4).

**Table 4:** The angle between the normal vector of each semicircular canal plane and the mean normal vector of the semicircular canal plane.

Normal vector	Mean (°)	SD
Left_A	6.8	4.19
Left_P	7.05	3.9
Left_L	9.31	4.63
Right_A	6.92	3.39
Right_P	7.97	4.82
Right_L	9.54	4.3

P, posterior semicircular canal; A, anterior semicircular canal; L, lateral semicircular canal.

## 4 Discussion

The spatial attitude of the semicircular canals, which refers to their shape and orientation in space, is crucial for examining vestibular function, and diagnosing and treating benign paroxysmal positional vertigo (BPPV). Regarding the angles between the posterior, lateral, and anterior semicircular canals on the same side, numerous related studies have been conducted, and these canals were generally considered to be perpendicular to one another (25).

Some studies have also measured the angle between the bilateral semicircular canals, especially the conjugated ones, or the angle between a semicircular canal and the coordinate plane (13, 21, 26). However, only a few studies have fully measured the spatial orientation of the semicircular canals within the cranial spatial coordinate system. Due to factors such as data quality and measurement methods, the data from various studies on the spatial attitude of semicircular canals are inconsistent or even self-contradictory.

Currently, understanding the angles between the three semicircular canals on the same side only helps us grasp the shape of unilateral semicircular canals. Even with more knowledge about the spatial relationship between bilateral semicircular canals, much remains to be understood about the overall spatial orientation of the semicircular canals. While some studies did not establish a body space coordinate system, they indirectly studied the spatial orientation of the semicircular canals by calibrating the spatial orientation of the bony semicircular canals with recognized spatial attitude data. So far, there has been a lack of high-quality research about semicircular canal orientation based on large datasets (16, 27).

Knowing only the angle between the three semicircular canals on the same side can aid in understanding the morphology of the unilateral semicircular canals. However, it is insufficient for comprehending the spatial relationship between bilateral semicircular canals and the overall spatial orientation of the semicircular canals. In some studies, although no spatial coordinate system was established, the spatial direction of the semicircular canals was indirectly studied by calibrating their spatial direction and the recognized spatial orientation data (20).

The method of establishing the semicircular canal plane is the most critical factor affecting the quality of related research. A simple approach is to define the plane using three points, but manual error is significant. The error can be minimized by utilizing multi-point coordinates of the semicircular canal and fitting a plane equation. Bisecting the semicircular canal plane is also a common method, but it is highly subjective. Using computer technology to automatically obtain the fitting plane of the semicircular canal centerline can minimize manual error, but it is also affected by the calculation method and range of the centerline. To obtain the plane equation of the semicircular canal, complete spatial attitude information is required. It should be noted that according to the equation of the semicircular canal plane, the unit normal vector can be obtained, and its spatial coordinate value is related to the head position and the selected spatial coordinate system. To ensure the comparability of research data, the spatial coordinate system and the direction of the unit normal vector should be systematically obtained. The normal vectors of the semicircular canal planes will vary from study to study because of the different definitions of the spatial coordinate system used, with the X, Y, and Z axes representing the normal vectors of the different planes.

In this study, the *X*-axis is the normal vector of the lateral plane, with its positive direction pointing upward; the *Z*-axis is the normal vector of the sagittal plane, with its positive direction pointing rightward; and the *Y*-axis is the normal vector of the coronal plane, with its positive direction pointing from inward to outward.

The establishment of a spatial coordinate system is a prerequisite for measuring the spatial direction of a semicircular canal. Commonly used three-dimensional coordinate systems, such as the Frankfurt coordinate system and the Reid coordinate system, are determined based on skeletal landmark points (20, 27). However, research on the spatial orientation of the semicircular canal has been limited due to insufficient scanning range or the difficulty in identifying skeletal landmark points and establishing spatial coordinate systems in MRI scans. It has been observed that the plane formed by the top of the common crus and the lowest point of the eyeball is parallel to the Frankfurt plane. This observation opens up the possibility of establishing a spatial coordinate system through the semicircular canal and the eyeball, providing a particularly suitable method for the automatic construction of a standard spatial coordinate system.

This paper achieved the following items: (1) An object recognition-based deep learning algorithm that achieved precise and rapid segmentation of the semicircular canals, providing a powerful solution for real-time automatic segmentation. (2) Utilizing semicircular canal and eyeball information to establish a spatial coordinate system thereby resolving the issue of lacking a spatial coordinate system in MRI research. (3) A three-dimensional skeletonization algorithm that obtained the centerline of the semicircular canal, enabling point-by-point analysis and identification of each canal.

Compared to previous studies, this study has a larger data volume of 115 cases. The degree of automation is higher, not only adopting deep learning for automated segmentation of semicircular canals and eyeballs, but also realizing intelligent measurement of the spatial orientation of the semicircular canals. The measurement accuracy is improved by not only adopting the 3D skeletonization algorithm to acquire the centerline of semicircular canals, but also further enabling point-by-point analysis of the centerline and identification of isolated and invalid points.

The Vascular Modeling Toolkit (VMTK) is a comprehensive suite of libraries and tools designed for the 3D reconstruction, analysis, and visualization of vascular structures derived from medical imaging data. VMTK has been employed in prior research to extract the centerline of semicircular canals, a process that necessitates a particular runtime environment for execution (28). This algorithm is programmed in Python, allowing it to run on radiologists’ computers and have a wider scope of application. The final established mathematical model of the spatial attitude of semicircular canals exhibits good left–right symmetry, which is important for understanding their spatial orientation. The results confirmed that the left and right lateral semicircular canals, as well as the posterior semicircular canal on one side and the anterior semicircular canal on the opposite side, were located in the same plane; the angle between the posterior semicircular canals and the sagittal plane was close to 45 degrees. However, the study showed that the angle between the lateral semicircular canals and the lateral plane was about 10 degrees, which was smaller than reported in the literature. The possible reason for this difference is related to the range of points taken, as this study used the full segmentation of the centerline of the lateral semicircular canal.

The limitation of this paper is that only plane analysis is done, but no surface analysis is done. The spatial normal vector of each segment is measured, and only the semicircular canal and the center line are fitted by plane. In the future, we can consider the range of points, and the fitting of the whole semicircular canal to the plane, or we can only consider the fitting of a certain semicircular canal.

## 5 Conclusion

This study presents a novel approach for intelligent understanding and measurement of the spatial orientation of semicircular canals using cranial MRI data. By integrating deep learning-based segmentation, 3D skeletonization, and point characteristic analysis, we have developed a robust and automated method for extracting and analyzing the centerlines of the semicircular canals. The nnDetection target recognition segmentation algorithm achieved high Dice values, demonstrating its effectiveness in accurately segmenting the semicircular canals and eyeballs. The computed direction angles of the unit normal vectors for the anterior, lateral, and posterior semicircular canal planes provide valuable insights into their spatial orientation. Furthermore, our approach introduces a standardized spatial coordinate system based on the top of the common crus and the bottom of the eyeballs, enabling consistent and reliable measurements across subjects. The utilization of curvature and torsion features allows for intelligent selection of representative points along the centerlines for plane fitting, resulting in a more accurate representation of the semicircular canals’ spatial orientation. The real-time automated understanding and measurement of the spatial orientation of semicircular canals achieved in this study have significant implications for vestibular research and clinical applications. It provides a solid foundation for personalized diagnosis and treatment optimization of vestibular disorders. Moreover, this work establishes essential tools and a theoretical basis for future investigations into vestibular function and related diseases.

In conclusion, our intelligent approach offers a comprehensive and efficient solution for analyzing the spatial orientation of semicircular canals using cranial MRI data. The methodology and findings presented in this study contribute to advancing our understanding of the vestibular system and pave the way for improved diagnostic and therapeutic strategies in the field of vestibular medicine.

## Data Availability

The original contributions presented in the study are included in the article/supplementary material, further inquiries can be directed to the corresponding author.

## Footnotes

^1^https://github.com/MIC-DKFZ/nnDetection
